# Autism Spectrum Disorder in Hypothalamic Hamartoma: Early Developmental Trajectory, Symptom Profile, and Relevance for Autism Research

**DOI:** 10.1155/crpe/3750797

**Published:** 2026-11-04

**Authors:** Pierre Augé, Charlotte Mansat, Geoffroy Delplancq, Dinane Samara-Boustani, Marie-Joëlle Orêve, Mario Speranza

**Affiliations:** ^1^ CESP Inserm Versailles Saint-Quentin-en-Yvelines University (UVSQ) Paris-Saclay University Versailles France inserm.fr; ^2^ PEDIATED–Child and Adolescent Psychiatry Unit Versailles Hospital Versailles France; ^3^ Department of General Pediatrics Paris-Saclay Hospital Orsay France; ^4^ Department of Pediatric Neurology Bicêtre Hospital (AP-HP) Le Kremlin-Bicetre France; ^5^ Constitutional Genetics Unit Versailles Hospital Versailles France; ^6^ Department of Paediatric Endocrinology Diabetology Gynaecology Necker-Enfants–Malades University Hospital (AP-HP) Paris France

**Keywords:** autism, case report, epilepsy, hypothalamic hamartoma, neurodevelopment

## Abstract

Focal brain lesions occurring during critical developmental periods can shed light on the neural substrates underlying autistic symptoms. Hypothalamic hamartomas (HH) are rare congenital malformations that are typically characterized by pharmacoresistant epilepsy, endocrine disturbances, and behavioral or psychiatric disorders. Although autistic symptoms have occasionally been reported in children with HH, they remain poorly described and are rarely documented with standardized diagnostic tools. We present a case report of a 3‐year‐old boy with HH who was followed from 2 years of age. The report combines detailed early neurodevelopmental history, neuroimaging data, and standardized autism assessments. The child exhibited global developmental delay, early intense sensory features, and marked social communication deficits. The first developmental difficulties preceded the onset of seizures. At 29 months of age, magnetic resonance imaging identified a small intrahypothalamic hamartoma. At 36 months, the Autism Diagnostic Observation Schedule‐2 and the Autism Diagnostic Interview‐Revised confirmed the diagnosis of autism spectrum disorder (ASD). A comparison with 3 subjects with ASD matched for sex, chronological age, and developmental age showed that the child presented less severe deficits in sharing enjoyment and less intense unusual preoccupations than age‐matched autistic peers. This case highlights the importance of systematic ASD screening in children with HH, allowing timely referral to evidence‐based early interventions. Moreover, HH may offer a valuable framework for investigating early neurodevelopmental mechanisms associated with ASD, while highlighting the potential contribution of the hypothalamus to early autism mechanisms.

## 1. Introduction

Autism spectrum disorder (ASD) is defined by persistent impairments in social communication alongside restricted and repetitive patterns of behavior. Lesional models of ASD provide important insights into its neural substrates. Among these, tuberous sclerosis complex (TSC) represents one of the best‐characterized models [1]. Similarly, cerebellar lesions from diverse etiologies can produce autistic‐like features, supporting an increasingly recognized role of the cerebellum in social cognition beyond motor control [2]. Early acquired brain injuries, such as perinatal stroke, further illustrate how structural damage during critical developmental periods may lead to autistic features [3]. Brain tumors arising early in life also provide natural models for examining the consequences of focal lesions during brain maturation, highlighting the importance of developmental timing and neuroplasticity.

Hypothalamic hamartomas (HH) are very rare benign malformations of the tuber cinereum with an estimated prevalence of 1 in 200,000 [4]. They can occur sporadically or as part of a syndromic presentation, the most common being Pallister–Hall syndrome [5]. The symptomatology of HH includes a triad of drug‐resistant epilepsy, central precocious puberty, and a wide range of behavioral or psychiatric disturbances [4, 6]. Two main anatomical subtypes are described: intrahypothalamic hamartomas (IHH) and parahypothalamic hamartomas (PHH).

Epilepsy in the context of HH typically begins at an early age, with a mean onset around 18 months, and affects up to 90% of patients [7]. Gelastic seizures, characterized by brief, unprovoked episodes of inappropriate laughter, are highly suggestive of HH [4] and are frequently followed by focal or secondarily generalized tonic–clonic seizures [7]. Psychiatric manifestations remain insufficiently documented due to the rarity of this malformation, despite evidence that more than half of patients develop at least one psychiatric disorder [8]. Rage attacks and heteroaggressive outbursts have been described as typical externalizing symptoms in this population [9].

Neurodevelopmental disorders (NDD) may occur in association with HH. Intellectual developmental disorder or global developmental delay occurs in more than half of cases and is influenced by lesion size, age at seizure onset, and seizure severity [6, 8]. ASD has been reported in approximately 15%–18% of children with HH [7, 10], and 34% of these children present social communication difficulties. Approximately 30% show attentional or executive impairments [10, 11], and 21% display language impairment [10]. Most of the time, autistic‐like symptoms precede seizure onset and appear to be independent of the anatomical subtype of the hamartoma [10]. Some authors have nevertheless argued that developmental difficulties may be secondary to epilepsy rather than directly caused by the lesion [12, 13]. Only one detailed pediatric case using standardized autism assessment tools has been published to date [14]. Importantly, the early developmental trajectory and specific autism profile of children with HH remain undescribed, and HH has not yet been explored as a model for studying early neurobiological mechanisms of ASD.

The present report describes a 3‐year‐old boy with IHH who was enrolled in neurodevelopmental follow‐up from an early stage. Written informed consent for publication of this case report was obtained from the child’s legal representatives. We documented his early developmental trajectory, seizure history, neuroimaging findings, and the results of standardized autism assessments. Through this case, we aim to describe the developmental trajectory and autistic phenotype associated with IHH and to consider HH as a framework for investigating early neurobiological mechanisms of ASD.

## 2. Case Description

### 2.1. Perinatal and Developmental History

The patient is the third child of nonconsanguineous Caucasian parents who each have a history of thrombosis secondary to a factor V Leiden mutation. The pregnancy was complicated by insulin‐treated gestational diabetes, a SARS‐CoV‐2 infection with pulmonary embolism at 30 weeks’ gestation requiring full‐dose anticoagulation and threatened preterm labor that was treated with atosiban. Prenatal ultrasound examinations were normal. Delivery was induced at 37 weeks and 2 days. The newborn weighed 3.1 kg, measured 49 cm in length, had a head circumference of 35.5 cm, and had a total Apgar score of 10/10 at 1 and 5 min. The neonatal period was notable for torticollis that interfered with breastfeeding, and a simple jaundice treated with phototherapy. No hospitalization was required beyond routine care.

Motor and language development was delayed. The patient achieved head control at 3 months, sitting posture at 10 months, quadrupedal mobilization at 14 months, and walked independently at 19 months. He began to babble at 12 months and produced his first words at 18 months. At 23 months, he had a vocabulary of about 5 words (Figure 1).

**FIGURE 1:**
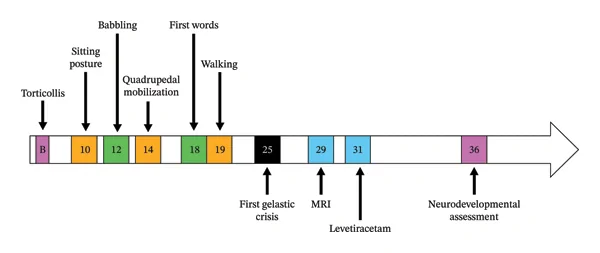
Timeline of developmental trajectory of the patient (age in month), from his developmental history. Orange: motor development. Green: language development. Black: seizure. Blue: care. B: birth. MRI: magnetic resonance imaging.

At 24 months, the patient showed a global motor and language delay, sensory features such as visual seeking and hand flapping, and deficits in nonverbal communication and self‐injurious behavior consisting of head banging. He was then referred for multidisciplinary care, including psychomotor therapy, speech therapy, child psychiatry, and neurology.

At 31 months, clinical observation revealed fluctuating eye contact, absence of pointing, lack of symbolic play and imitation, solitary play with a short attention span, and prominent sensory features. At 36 months, he underwent standardized assessments in our expert autism center.

### 2.2. Seizure Onset and Imaging

At 20 months, the patient exhibited episodes suggestive of generalized seizures. Electroencephalography (EEG) showed abundant generalized slow spike–wave discharges. By 23 months, generalized seizures had resolved and were replaced by isolated rictus episodes. At 25 months, the presentation evolved into clear gelastic seizures. The EEG then showed resolution of generalized abnormalities with a few focal slow waves.

At 29 months, brain magnetic resonance imaging (MRI) revealed an ovoid mass measuring 9.5 by 8.4 by 7.3 mm located above the right mammillary body and protruding into the third ventricle (Figure 2). This appearance was consistent with IHH. Levetiracetam was introduced at 31 months for persistent seizures and titrated up to 55 mg/kg/day, and the child has remained seizure‐free since then.

**FIGURE 2:**
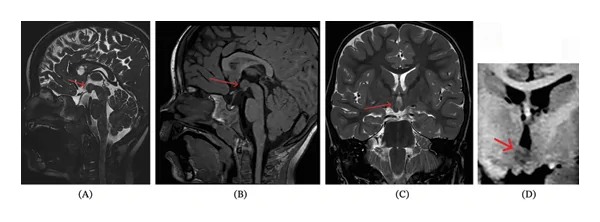
Cerebral MRI of the patient in favor of HH. Sagittal T2 (A), sagittal T1 (B), coronal T2 (C), and coronal T1 (D). Red arrows show the IHH.

Genetic analyses (fragile X testing, CGH array, and trio‐exome sequencing), comprehensive blood investigations (complete blood count, electrolytes, creatinine, liver function tests, ammonia, lactate, CPK, Metaglut‐1, and plasma amino acids), and a full endocrine evaluation performed at a specialized pediatric endocrinology service (including pituitary function, but not oxytocin) all yielded normal results.

### 2.3. Results of Standardized Developmental Assessments

The patient was seizure‐free throughout the period of evaluation conducted in our expert autism center.

On the Autism Diagnostic Interview‐Revised (ADI‐R), parents reported an absence of pointing, lack of pretend play, abnormal gaze pattern during the social interactions, and infrequent gestural communication. Repetitive behaviors included hand mannerism, flapping, intense sensory features, and repetitive use of objects (Figure 3A).

**FIGURE 3:**
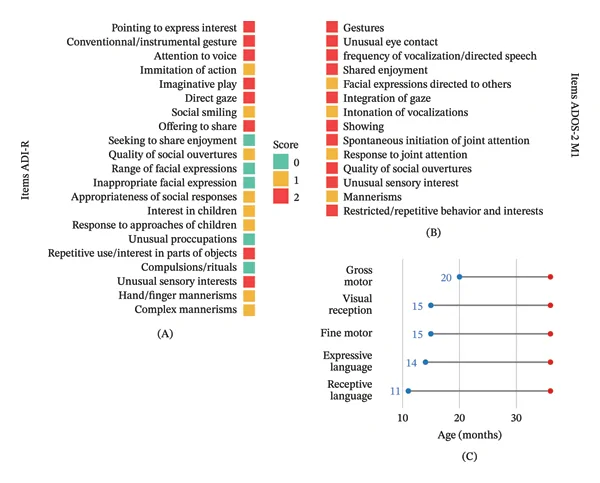
Results of the patient’s autism assessment. ADI‐R result by item (A). ADOS‐2 module 1 result by item (B). MSEL results: the red point corresponds to the chronological age (36 months), and the blue point to the developmental age (C). Items from the ADI‐R and the ADOS‐2 are coded according to severity and frequency, where 0 represents behavior within expectations of typical development, 1 represents mild or atypical manifestation, and 2 represents marked abnormality. Green: 0, orange: 1, and red: 2. ADI‐R: Autism Diagnostic Interview‐Revised. ADOS‐2: Autism Diagnostic Observation Schedule‐2. MSEL: Mullen Scales of Early Learning.

On the Autism Diagnostic Observation Schedule‐2 Module 1 (ADOS2‐M1), eye contact remained inconsistent, and his facial expression was neutral. Turn‐taking was absent, and sharing of enjoyment was only fleeting. Joint attention was lacking. Vocalizations were rare, but imitation skills were beginning to emerge. Restricted behaviors included hand flapping, objects alignment, and prolonged visual inspection of objects (Figure 3B).

The Mullen Scales of Early Learning (MSEL) scores indicated a developmental age of 20 months in gross motor, 15 months in visual reception and fine motor, 14 months in expressive language, and 11 months in receptive language domains (Figure 3C).

To provide an observational description of how the patient’s IHH might relate to his autistic profile, we descriptively compared the ADI‐R, at the item level, with 3 autistic subjects without epilepsy matched for sex, chronological age (patient age ± 3 months), and developmental age, as assessed by MSEL subscales (patient developmental quotient ± 0.2). Written informed consent was obtained from the primary caregivers of all participating children. The sociodemographic and symptomatic characteristics of the patient and these subjects are shown in Table 1. The patient showed lower scores than the three children included in the comparison group for item 54 (“sharing enjoyment”) and item 67 (“unusual preoccupations”) (Figure 4).

**TABLE 1:** Sociodemographic, neurodevelopmental milestones and symptomatic characteristics of the patient and the 3 control subjects.

Subjects		Patient	1	2	3
Age (months)		36	39	35	33

Total Apgar score (/10, 1 min)		10	NA	10	9

First words (age in months)		18	NV	NV	12

Walking (age in months)		19	16	16	13

Developmental age (months)	GM	20	17	21	14
	FM	15	22	21	10
	VR	15	18	16	10
	EL	14	16	13	8
	RL	11	9	10	6

ADI‐R	Social Affects	9	16	15	15
	RRB	6	7	8	5
	Imitation, gesture, play	9	11	12	8

ADOS‐2	Social Affects	18	NA	NA	NA
	RRB	6	NA	NA	NA
	CSS	9	8	7	9

Abbreviations: ADI‐R, Autism Diagnostic Interview‐Revised; ADOS‐2, Autism Diagnostic Observation Schedule‐2; CSS, Calibrated Severity Score; EL, expressive language; FM, fine motor; GM, gross motor; NA, nonavailable; NV, nonverbal; RL, receptive language; RRB, restricted repetitive behavior; VR, visual reception.

**FIGURE 4:**
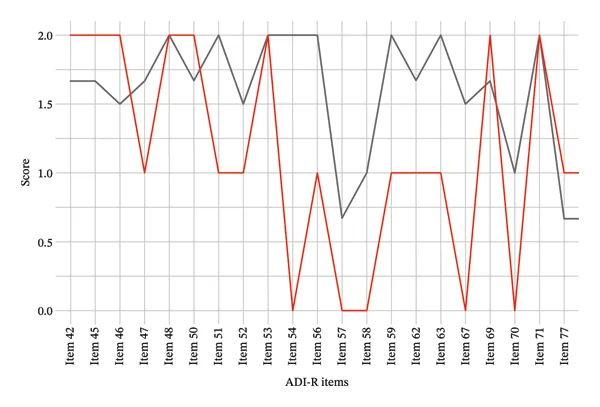
Profile of autistic symptoms based on items from the ADI‐R “single word” algorithm. The *x*‐axis represents the individual ADI‐R items, and the *y*‐axis represents the corresponding ADI‐R item scores. The patient (red line) is compared to the mean of the 3 subjects (gray line) for each item.

## 3. Discussion

Here, we present a case of ASD with global developmental delay in a young 36‐month‐old boy with IHH. Detailed medical history and regular early follow‐up allow us to accurately describe his early developmental trajectory during his first 3 years of life. In addition, the assessment carried out at our expert autism center allows us to describe his current autistic profile in detail.

### 3.1. Clinical and Neurodevelopmental Features

The first developmental particularities of the patient emerge during the first year of life. He is described as a quiet child, with babbling starting around 12 months. His early developmental trajectory is further marked by motor difficulties (walking at 19 months) and delayed language acquisition (first words around 18 months). In this respect, his early developmental trajectory shows many similarities with an autistic‐type trajectory. Indeed, from 6 months of age, children later diagnosed with ASD or at high risk of ASD are characterized by poorer performance in gross motor skills, fine motor skills, and visual reception (ability to recognize, differentiate, and interpret visual information such as shapes, colors, or images) compared to low‐risk children [15, 16]. At 9 months, language delay can be found in children who will be diagnosed with ASD [17].

Autistic symptomatology should be interpreted in relation to the child’s developmental level, given that several ASD symptom measures show differential item functioning across nonverbal mental age [18]. Therefore, we compared our patient with three age‐, sex‐, and developmental level‐matched children with ASD. Overall, the patient’s symptom profile was broadly similar to that of the matched children, consistent with findings in children with ASD and TSC, the genetic lesional epilepsy syndrome phenotypically closest to IHH [19, 20]. Jeste et al. reported that the ASD symptom profile of children with TSC was broadly similar to that of children with nonsyndromic ASD, in a cohort with an age range comparable to that of our study [19]. In this study, social smiling, showing, and joint attention appeared relatively better preserved in the TSC‐ASD group [19], whereas our patient showed relative strengths in sharing enjoyment and unusual preoccupations. These differences may partly reflect differences in assessment methods, as our comparison was based on the ADI‐R whereas Jeste et al. used the ADOS. Further studies directly comparing the autistic profiles associated with TSC and HH would, therefore, be of particular interest for understanding the neurobiological basis of autistic symptoms in lesional epilepsy syndromes.

It is also essential to examine the patient’s symptomatic profile in the context of ASD–epilepsy comorbidity. Such profiles are characterized by more pronounced repetitive object use, heightened sensory sensitivities, and greater global motor coordination difficulties [21]. This pattern is consistent with our patient, who exhibits particularly intense sensory features and marked motor delay.

Finally, the patient’s autistic profile should also be interpreted in the context of his ongoing treatment. The ADOS‐2 was performed while he was receiving levetiracetam, a drug known to be associated with behavioral side effects, particularly irritability, in some children [22], and occasionally with reversible autistic‐like regression [23]. Importantly, the patient’s developmental difficulties had already emerged well before levetiracetam was introduced, and he did not display irritability or agitation during the assessment. These observations argue against a major influence of levetiracetam on his behavioral profile at the time of assessment, although a medication effect cannot be completely excluded.

### 3.2. Neurobiological Hypotheses

It is important to analyze the patient’s case in the context of the comorbid ASD and epilepsy. Several studies show that the onset of epilepsy can both precede and follow the diagnosis of NDD [24]. Epilepsy and ASD may arise from shared neurodevelopmental mechanisms, such as dysregulation of the balance between neuronal excitation and inhibition [25]. Conversely, early epilepsy could also affect synaptic plasticity, predisposing the individual to neurodevelopmental difficulties [26]. In the patient, the first episodes suggestive of seizures appeared around 20 months of age, after the first developmental concerns. This temporal pattern supports the hypothesis that the IHH itself can exert a direct neurodevelopmental effect rather than the autistic features being solely secondary to epilepsy. However, brain MRI was performed only at 29 months and, therefore, cannot determine the potential effects of the lesion at an earlier age. An alternative explanation is that subtle or unrecognized epileptiform activity may have been present before the onset of clinically apparent seizures and may have influenced neurodevelopmental trajectories during critical periods of brain maturation. Recurrent epileptiform discharges, even in the absence of overt clinical seizures, may interfere with the development and organization of neural networks involved in cognition, social communication, and behavior [27]. In patients with HH, where epileptogenesis may evolve progressively before the emergence of clinically recognizable seizures [28], such subclinical activity could, therefore, potentially contribute to early neurodevelopmental abnormalities [12, 13].

Several other mechanisms may explain the association between HH and ASD. The hypothalamus plays a central role in social behavior and neuroendocrine regulation, including oxytocin pathways that modulate social skills [29]. Disruption of these circuits by a HH could alter early social motivation, favoring the emergence of an autistic phenotype. In our patient, endocrine investigations, including pituitary function, were within normal limits.

Moreover, we can hypothesize that thalamic involvement could potentially contribute to sensory features in ASD, as the role of the thalamus in sensory processing has been supported by several neuroimaging studies [30], including studies performed during the first year of life [31]. There was no evidence of thalamic compression on MRI in our patient. Given the size and type of his HH, direct mechanical compression of the thalamus is, therefore, unlikely in this case, but such a mechanism could potentially be considered in patients with giant HH. Importantly, thalamic involvement does not necessarily require direct mechanical compression by the HH. The concept of secondary epileptogenesis suggests that an epileptogenic focus may progressively disrupt connected neuronal networks and promote the emergence of secondary epileptogenic foci [28]. Given the extensive connectivity between the hypothalamus and several thalamic nuclei [32], early epileptic network dysfunction could, therefore, alter thalamic circuits without structural compression. This provides a potential network‐level mechanism linking HH to thalamic dysfunction, although this remains a theoretical hypothesis requiring further investigation.

Finally, rather than a direct cascade where the HH or epilepsy strictly drives autistic features, a shared early neurodevelopmental mechanism could explain this triad (HH, epilepsy, and ASD). Disruptions in key developmental signaling pathways, such as the Sonic Hedgehog (Shh) pathway or mammalian target of rapamycin (mTOR) signaling [20], or early postzygotic somatic mutations affecting neural progenitor cell migration and synaptogenesis [33], could simultaneously induce the ventral midline malformation (HH [34, 35]), alter cortical circuit wiring underlying ASD [36, 37], and generate an epileptogenic network [37, 38]. In our patient, the normal genetic analyses do not exclude the possibility of a somatic mutation.

### 3.3. Limitations and Implications

This case report has several limitations. Being a single case, the results may not be generalizable to all children with HH. Long‐term follow‐up after potential surgical intervention will be informative to determine whether autistic symptoms improve, remain stable, or worsen over time. Finally, the comparison with only three children with ASD is purely descriptive and should be interpreted as an anecdotal, hypothesis‐generating observation rather than as a representative or generalizable finding. However, to our knowledge, our study is the first to precisely describe the early developmental trajectory of a child with HH.

This case has several important clinical implications. Children with HH should undergo systematic screening for ASD using validated tools regardless of seizure status. Early behavioral intervention may be warranted even before epilepsy surgery. Multidisciplinary follow‐up including neurology, endocrinology, developmental pediatrics, and child psychiatry is essential to address the complex needs of these children. By focusing on psychiatric and behavioral manifestations, which are among the main concerns reported by families [34], this study also addresses one of the key priorities for future research identified by the expert committee of the International Symposium on Hypothalamic Hamartoma [34].

Finally, this case has theoretical implications. It highlights the potential contribution of hypothalamic dysfunction to early neurodevelopment and the emergence of autism‐related features. Despite the rarity of HH, longitudinal and multicenter studies may provide valuable opportunities to investigate the neurobiological mechanisms underlying ASD. Systematic assessment of oxytocin levels in children with HH could help clarify its potential contribution to neurodevelopmental features and provide an informative model for studying oxytocin‐related mechanisms in ASD. More broadly, comparing the ASD phenotype associated with genetic or lesional syndromes with idiopathic ASD may help disentangle syndrome‐specific mechanisms from those underlying ASD more broadly, an approach supported by previous systematic review and meta‐analysis [39].

## 4. Conclusion

We report the case of a young boy with ASD associated with IHH, in whom developmental concerns clearly preceded the onset of seizures. Standardized assessments confirmed the diagnosis of ASD with social communication deficits and intense sensory features. This case underscores the importance of early neurodevelopmental evaluation in children with HH and contributes to a better understanding of the complex interplay between hypothalamic malformations, epilepsy, and ASD. By documenting the developmental trajectory from birth and using standardized tools, this report provides new evidence that may guide clinical practice and future research.

## Funding

No funding was received for this manuscript.

## Conflicts of Interest

The authors declare no conflicts of interest.

## Supporting Information

Additional supporting information can be found online in the Supporting Information section.

## Supporting information


**Supporting Information** CARE checklist.

## Data Availability

The data that support the findings of this study are available on request from the corresponding author. The data are not publicly available due to privacy or ethical restrictions.
